# Use, applicability, and dissemination of patient versions of clinical practice guidelines in oncology in Germany: a qualitative interview study with healthcare providers

**DOI:** 10.1186/s12913-024-10626-8

**Published:** 2024-03-04

**Authors:** Sarah Wahlen, Jessica Breuing, Monika Becker, Stefanie Bühn, Julia Hauprich, Nadja Könsgen, Nora Meyer, Susanne Blödt, Günther Carl, Markus Follmann, Stefanie Frenz, Thomas Langer, Monika Nothacker, Corinna Schaefer, Dawid Pieper

**Affiliations:** 1 Institute for Research in Operative Medicine (IFOM), Witten/Herdecke University, Cologne, Germany; 2 Institute for Medical Knowledge Management c/o Philipps University Marburg, Association of the Scientific Medical Societies in Germany, Marburg/Berlin, Germany; 3 German Prostate Cancer Support Group, Bonn, Germany; 4 Office of the German Guideline Program in Oncology (GGPO), c/o German Cancer Society, Berlin, Germany; 5 Frauenselbsthilfe Krebs–Bundesverband e.V., Bonn, Germany; 6 German Agency for Quality in Medicine, Berlin, Germany; 7 Faculty of Health Sciences Brandenburg, Brandenburg Medical School (Theodor Fontane), Institute for Health Services and Health System Research, Rüdersdorf, Germany; 8 Centre for Health Services Research, Brandenburg Medical School (Theodor Fontane), Rüdersdorf, Germany

**Keywords:** Patient version of clinical practice guidelines, Qualitative interviews, Healthcare providers, Oncology, Patient information, Consumer health information

## Abstract

**Background:**

People with cancer have high information needs; however, they are often inadequately met. Patient versions of clinical practice guidelines (PVGs), a special form of evidence-based information, translate patient-relevant recommendations from clinical practice guidelines into lay language. To date, little is known about the experience of PVGs from healthcare providers’ perspective in healthcare. This study aims to investigate the use, applicability, and dissemination of PVGs in oncology from the healthcare providers’ perspective in Germany.

**Methods:**

Twenty semi-structured telephone interviews were conducted with oncological healthcare providers in Germany between October and December 2021. Interviews were recorded and transcribed verbatim. Mayring’s qualitative content analysis with MAXQDA software was utilised to analyse the data.

**Results:**

A total of 20 healthcare providers (14 female, 6 male), mainly working as psychotherapists/psycho-oncologists and physicians, participated. Most participants (75%) were aware of the existence of PVGs. The content was predominantly perceived as comprehensible and relevant, whereas opinions on the design and format were mixed. The perceived lack of up-to-date information limited participants’ trust in the content. Most felt that PVGs positively impact healthcare owing to the fact that they improve patients’ knowledge about their disease. Additionally, PVGs served as a guide and helped healthcare providers structure physician–patient talks. Healthcare provider’s unawareness of the existence of PVGs was cited as an obstructive factor to its dissemination to patients.

**Conclusion:**

Limited knowledge of the existence of PVGs among healthcare providers, coupled with alternative patient information, hinders the use and dissemination of PVGs in healthcare. However, the applicability of PVGs seemed to be acceptable owing to their content and good comprehensibility, especially with respect to physician–patient communication.

**Supplementary Information:**

The online version contains supplementary material available at 10.1186/s12913-024-10626-8.

## Background

Several studies have shown that, particularly in the field of oncology, patients’ need for medical information is high but often unmet [1–4]. A recent overview of systematic reviews found that hospitals provide inadequate health information to patients, which can lead to poor quality patient care and low patient satisfaction [5]. In 2001, the Institute of Medicine (IOM) issued recommendations for achieving high quality care in six areas of healthcare, including patient-centredness [6]. The emphasis was placed on the patient as a complex individual rather than on a simple treatment approach. The IOM recommended six aspects of patient-centred healthcare, notably the provision of information, communication and patient education [6]. Providing health information and advice to patients enables healthcare providers to meet patients’ requirement of information needs and meet the IOM standards for patient-centred care [6], as well as supporting patients’ autonomy and empowering them to engage in healthier behaviour [7, 8]. To fulfil patients’ information needs, healthcare providers need to familiarise themselves with the relevant health information and distribute it. However, they may only recommend certain health information that they subjectively consider valid. Efforts are already being made to improve the communication skills of healthcare professionals, also in the field of oncology [9]. These efforts focus on how to best communicate information to patients, but do not specifically include evidence-based patient information [9]. Clinical practice guidelines (CPGs) provide evidence-based recommendations and up-to-date knowledge about the prevention, diagnostic procedures, treatments or follow-up policies of specific medical conditions [10]. CPGs mainly address healthcare providers and help them make decisions regarding appropriate patient care [10–12]. However, their content is written in medical language and might be difficult for laypersons to understand. Moreover, the general concept of CPGs (i.e. methods/procedures to develop recommendations) is difficult for many patients and non-professionals to comprehend [13, 14]. Although CPGs are mainly designed for healthcare providers, patients, their relatives, friends, and other non-professionals involved in patient care sometimes use them as information sources [15, 16]. To make the concepts and content of CPGs more accessible to patients and other laypersons, several international organisations have developed patient versions of CPGs (PVGs) and translated their recommendations into common speech that the average layperson would understand [17]. The German Guideline Program in Oncology (GGPO) develops PVGs in the field of oncology to address the information needs of oncological patients in Germany. To date, the GGPO has provided several PVGs for various oncological diseases. These PVGs provide information on the diagnosis, treatment and follow-up care and address various patient populations in different disease stages (early/metastazised). In addition, the GGPO provides PVGs for cross-sectional oncological topics, such as psycho-oncology, early detection, supportive care and palliative care. The PVGs of the GGPO are distributed online (in PDF format) and as print brochures. PDF versions are accessible via the GGPO and German Cancer Aid websites [18, 19], and brochures can be ordered through German Cancer Aid [19]. Both options are free of charge. A list of all existing oncological PVGs in Germany can be found on the GGPO website [18].

As healthcare providers play a key role in the dissemination of health information, it is important to know their assessment of PVGs and adapt information materials accordingly. However, to the best of our knowledge, information regarding healthcare providers’ perspectives on oncological PVGs is scarce. This study aims to investigate the use, applicability, and dissemination of PVGs in oncology from the perspective of healthcare providers in Germany.

## Methods

### Design

This study is part of a large multi-phase study (AnImPaLLO project) investigating the (inter-)national role and applicability of PVGs in oncology in order to derive recommendations for the development, dissemination, and implementation of PVGs in Germany. The study was conducted in two main modules: Module 1 investigated the applied methods and approaches on development and dissemination of PVGs, and Module 2 conducted separate semi-structured interviews and joint focus groups with national healthcare providers and patients to focus on a national perspective on the implementation and dissemination of PVGs. Detailed information can be found in the protocol [20]. The project was set up in cooperation with relevant stakeholders (hereafter, project partners) that are involved in development of patient versions in Germany: the GGPO, the Association of the Scientific Medical Societies in Germany - Institute for Medical Knowledge Management (AWMF-IMWi), the German Agency for Quality in Medicine (ÄZQ), and two German self-help groups focusing on prostate cancer (Bundesverband Prostatakrebs Selbsthilfe [BPS]) and cancer in women (Frauenselbsthilfe Krebs–Bundesverband [FSH]).

The present study focuses on semi-structured interviews targeting the perspectives of healthcare providers in Germany. We followed the consolidated criteria for reporting qualitative research (COREQ) checklist [21] to report our study (Supplement 1: COREQ-checklist).

### Recruitment

Healthcare providers directly involved in the care of cancer patients (e.g. physicians, psycho-oncologists, nurses), aged 18 years or older and with sufficient knowledge of German language were recruited. There were two ways of recruitment. First, participants were recruited via an online survey to analyse their awareness and the role of PVGs in oncology. The survey was conducted between April and June 2021 by the AWMF-IMWi and was not part of the AnImPaLLO project [22]. After completing the survey, healthcare providers were asked whether they would like to take part in the AnImPaLLO interviews and, if interested, provided their email address for recruitment purposes. The authors then contacted the interested survey participants. Second, project partners published calls for study participation via the Internet (e.g. newsletters, websites, social media) or flyers. After creating a list of all existing centres using Microsoft Excel, we also contacted a randomised national sample of certified and non-certified oncology centres in Germany. A new numeration of the centres was created by assigning a random number to each centre using the RAND function, and the first 50 centres on the list were contacted. A central organisation (the German Cancer Society) certifies oncology centres and recognises inpatient and outpatient facilities that form a network (centre) to improve the treatment of oncology patients through cooperative efforts. Information on certification of oncology centres in Germany can be found on the OnkoZert website [23]. Relevant hospital units of certified and non-certified centres (e.g. outpatient clinic, psycho-oncology) were contacted by telephone to recruit medical providers who were directly involved in patient care. If the telephone approach was unsuccessful or impossible, the relevant hospital units were contacted via email. Moreover, we asked the participants whether they could pass on information about the study to colleagues to recruit more participants (snowball recruitment method). Recruitment ended when saturation was reached [24, 25], indicating no additional analytical themes.

### Data collection

Data were collected via telephone interviews with one author (MB), female researcher and, at the time, doctoral candidate at Witten/Herdecke University. The interviewer was trained in advance in qualitative interviews and analyses. The first contact between the participants and the interviewer occurred prior to the interviews when detailed information about the study (background, duration of the interview, and intention to publish the results), along with privacy statements, was provided to each participant. There was no relationship between the interviewer and participants. Participants were informed that she was a researcher at Witten/Herdecke University. The interview guide was designed prior to conducting the interviews but without any existing framework and consisted of three main sections: (1) general information, (2) general questions about PVGs, and (3) questions about specific PVGs, and was reviewed and modified by the project team (Supplement 2: interview guide). Two pre-test interviews were conducted, which did not result in any changes to the interview guide. Participants received one version of the PVG for discussion with the interviewer. PVG version selection was based on the oncological field in which the participants worked at or were the most involved. All interviews were conducted via telephone between October and December 2021 using a recording device. Field notes were not taken during the interviews. The interviewer joined the interview from the workplace or home office, and participants were free to choose a convenient place and timeframe. Consequently, the presence of other people cannot be ruled out. Repeat interviews were not conducted.

### Data processing

During the recording of the interviews, the author (MB) avoided bringing up personal details of the interviewees to prevent them from being recorded. An external agency was hired to transcribe the audio files of the semi-structured interviews verbatim. Subsequently, two authors (MB and SW) checked the quality of the transcripts and, if necessary, removed personal details to prevent any conclusions regarding individual participants. The final transcripts were then assigned IDs that were available to the researchers. Participants were not asked to provide feedback on their findings or transcripts.

### Data analysis

Based on the interview guide, data codes were developed and divided into 11 groups with several sub-codes: (1) general information about participants, (2) questions about PVGs in general and questions about specific PVGs, (3) general judgments, (4) design and presentation, (5) comprehensibility, (6) format, (7) trust in PVG, (8) content, (9) impact of patient version, (10) dissemination of PVG to the patient, and 11) perception of specific topics. The rules of code specification were defined for each code before analysing the interviews (Supplement 3: data coding system). Due to personnel changes within the project team, the interviewer (MB) was not available to conduct the interview analysis. Therefore, the analysis was performed by two other authors (SW and JB). MAXQDA software (version 2022) was used to perform the interview analysis according to Mayring’s content analysis method [26]. To structure the text into codes (categories) and sub-codes (sub-categories), both deductive (a priori defined data codes derived by the interview guide and used on the whole document) and inductive approaches (development of additional themes and sub-codes for the pre-existing material, used at the code level) were used. The interviews were analysed in a two-step process. First, two authors (SW and JB) coded five out of 20 transcripts independently and met to discuss and reach a consensus on the a priori defined data codes. Afterwards, they split the remaining sample of interviews and carried out the analysis independently with ongoing consultation meetings (deductive approach). Second, the final sample of data codes was split in half, and each author independently generated sub-codes for the selected data codes for the entire interview set. The sub-codes were presented, discussed, and agreed upon by the two authors during ongoing meetings (inductive approach). Further issues that arose during the independent coding process were discussed by the two authors during consultation meetings. Subsequently, the coding framework with the final categories and sub-categories was reviewed by a team of authors (SW, JB, SBl, and MN), and minor editorial modifications were made.

## Results

Overall, 36 healthcare providers showed an interest in participating in the study. A total of 16 participants (44%) did not participate, either because interviewers were unable to reach interested healthcare providers or because they were no longer working in the field of oncology. The remaining 20 healthcare providers participated in the semi-structured telephone interviews. Two of them were recruited via the initial AWMF-IMWi survey. Most participants worked as physicians (*n* = 5; 25%) or psycho-oncologists (*n* = 9; 45%) and in certified clinics (*n* = 12; 60%). Participants were predominantly female (*n* = 14; 70%), had a mean age of 51 years (range: 33–71), and an average of 15 years of professional experience with oncological patients (range: 3–31). Table 1 lists the participants’ characteristics. The average duration of the interviews was 34 min (range: 20–49 min).

Most participants were aware of the existence of PVGs (*n* = 15) and stated that they provided them to their patients in either print or online formats. A few participants reported not providing PVGs to patients unless specifically asked.

**Table 1:** Characteristics of Participants

ID	Sex	Age [years]		Profession	Professional experience in oncology [years]	Type of institution	Discussed PVG
1	Female		71	Psycho-oncologist	23	Medical practice	Breast cancer, metastasized
2	Female		45	Physician	12	Clinic (certification)	Breast cancer, metastasized
3	Female		43	Physiotherapist	16	Clinic (certification)	Breast cancer, metastasized
4	Male		54	Physician	26	Clinic (no certification)	Prostate cancer, metastasized
5	Female		51	Psycho-oncologist	5	Medical practice	Kidney cancer, metastasized
6	Male		61	Psycho-oncologist	10	Medical practice	Breast cancer, metastasized
7	Female		58	Psycho-oncologist	5	Clinic (certification)	Colon cancer, early
8	Female		62	Psycho-oncologist	15	Medical practice	Breast cancer, metastasized
9	Female		60	Psycho-oncologist	20	Clinic (no certification)	Supportive therapy
10	Female		53	Psycho-oncologist	2	Clinic (certification)	Psycho-oncology
11	Female		60	Nurse/medical assistant	31	Clinic (certification)	Prostate cancer, early
12	Male		33	Physician	n.i.	Medical practice	Prostate cancer, early
13	Female		57	Nurse/medical assistant	9	Clinic (certification)	Breast cancer, metastasized
14	Female		33	Nurse/medical assistant	12	Clinic (certification)	Breast cancer, metastasized
15	Male		34	Psycho-oncologist	7	Clinic (certification)	Psycho-oncology
16	Female		59	Physician	22	Clinic (certification)	Supportive therapy
17	Male		42	Psycho-oncologist	14	Clinic (certification)	Prostate cancer, metastasized
18	Male		47	Physician	16	Clinic (no certification)	Supportive therapy
19	Female		42	Nurse/medical assistant	21	Clinic (certification)	Colon cancer, early
20	Female		56	Nurse/medical assistant	3	Clinic (certification)	Supportive therapy

Abbreviations: n.i: no information; PVG: Patient version of clinical guidelines

In the study protocol, we planned a study population of approximately 25 participants [20]. However, the actual number of participants was lower (*n* = 20) because no additional results were obtained in the course of conducting the last interviews. Therefore, we assumed content saturation had been achieved and stopped recruitment.

### Impact on healthcare

Most participants felt that PVGs had a positive impact on healthcare for patients, healthcare providers, and their relationships. Some participants highlighted the positive impact of PVGs on patients’ confidence in treatment, as well as on their relatives and friends. Two participants expressed the following statements:

*‘So from a psycho-oncological point of view, I would say it can really give security because it gives clarity and information’. (ID05)*

*‘I believe that just as it is now, it is very helpful for […] the patient. […] If the spouse reads it, the girlfriend, whatever. Who then simply has this knowledge, in order to act as a stable person, vicariously convey hope, confidence […]’. (ID06*)

Several participants expressed that PVGs improved the patients’ knowledge of their disease, explaining that this could lead to targeted questions from patients during physician–patient talks and generally improved physician–patient communication.

According to the participants, PVGs not only hold a preparatory role for patients in terms of general communication with healthcare providers but also for healthcare providers involved in patient care. PVGs’ structured content helps participants refresh their medical knowledge and prepare for questions or content that might be interesting and important for patients. Simultaneously, wording in the PVGs serves as guidance for participants regarding word choice during conversations with patients. Many participants found the content of the PVGs to be comprehensible. Therefore, existing information needs could be met by the PVG. But some participants have said that patients´ information needs are different or may even be unknown or difficult for patients to understand. However, not all information needs seem to be met.

*‘And I believe that people can’t even formulate what information they need. Because it doesn’t occur to them that they have to lie in bed and vomit, and you’ve forgotten to put a spit bag in front of them. (…) They cannot demand this information. And this information is missing.’ (ID03)*.

But given the amount of information already included in the PVGs, an information overload should be avoided.

*‘And that’s such an overload of information that I think it’s sometimes too much for the condition the patients are in at the time.’ (ID03)*.

Thus, many participants would not include any further patient histories in the PVG, to avoid further increasing the volume of PVGs. Additionally, it was noted that self-help groups are a more suitable medium for sharing individual information.

According to a few participants who work as physicians, the reference to patient-relevant information in PVGs facilitates physician–patient talks, particularly considering the limited duration of these talks. However, some participants raised concerns about the impact of PVGs on physician–patient talks because patients may not perceive PVG content regarding treatment as recommendation but rather as a mandate. It was pointed out that this has not happened yet, but it could be a possible scenario. One participant stated:

*‘But if the patient who arrives with the guideline and says, “Now you have to do this and this”, it can lead to a problematic relationship’. (ID15)*

### Dissemination

A major barrier to the dissemination of PVGs is the lack of knowledge regarding their existence. One participant stated:

*‘What I think makes it difficult, is that they [PVGs] are little known. At least in my experience few people know about them’. (ID02)*

Other barriers mentioned were fear of hidden costs for ordering brochures and the number of other information brochures sent to clinics and hospitals from other providers. In contrast, some participants suggested that the unrequested delivery of PVGs to clinics and hospitals could improve awareness and, subsequently, the dissemination of PVGs. Furthermore, participants suggested that the cost neutrality of PVGs should be displayed more clearly.

In addition to the structural barriers listed above, individual-person-related barriers were mentioned. One participant noted language barriers or the intellectual capacity of the patients in this context.

*‘When a 60-year-old with a high degree of language barrier comes to me, I don’t hand him a guideline [PVG]. In addition, there are patients who […] don’t have the, let’s say, intelligence […] to be able to deal with such information’. (ID12)*

According to participants, healthcare providers, especially physicians, play the most important role in delivering PVGs to patients, followed by self-help groups and other information sources such as social media. In addition, participants were asked about the appropriate timing for handing over PVGs. While the answers varied, the majority found the time of diagnosis to be the most convenient. One physician explained as follows:

*‘When the diagnosis is made […]. Especially in the beginning, they [patients] need a lot of information and sometimes want to know a lot’. (ID14)*

In contrast, another participant reported that patients might experience emotional shock and distress immediately after diagnosis and recommended against early confrontation through PVGs. However, according to most participants, dissemination during treatment seemed appropriate.

Participants were asked about the influence of PVGs compared to other information sources in Germany. The majority compared PVGs to another source of information for oncological patients of German Cancer Aid called ‘Blaue Ratgeber’ in German, noting that while the former was more detailed, the latter had better distribution to hospitals and medical practices. Moreover, participants found PVGs and patient information from the German Cancer Aid to be complementary as they vary in detail. Examples of patient information of German Cancer Aid can be found on its website (https://www.krebshilfe.de/informieren/ueber-krebs/infothek/infomaterial-kategorie/die-blauen-ratgeber/).

### Other topics

Participants had mixed opinions on PVG designs. Some found that the colours (pastel-red/orange) were friendly and neutral, whereas others wanted more vivid colours to enliven the text. In addition, participants had mainly positive impressions of the graphics, info boxes, and the text structure, while the majority criticised the large volume of PVGs. The majority preferred the brochure format for PVGs. Furthermore, participants found that patient age played an important role in the preference for format as younger patients may prefer the PDF-version of PVGs while older patients preferred the brochure. According to the participants, the overall comprehensibility of PVGs was good owing to the restricted use of medical words in favour of plain language. Many participants were aware of medical recommendations and recognised them in the text. However, the majority assumed that the recommendations were not recognisable or comprehensible to patients. According to the majority, PVG content included important information, although some participants thought information about patient self-care or aspects of complementary medicine was lacking and criticised the lack of up-to-date information of certain aspects (e.g. medications). The lack of up-to-date information had influences on the perceived trust in the content. As an option for improvement, the participants suggested living guidelines, which aim to optimise the guideline development process by updating individual recommendations as soon as new relevant evidence becomes available [27]. Hence, the participants suggested that PVGs could also be adapted to their living status. Table 2 provides additional information.

**Table 2:** Additional results

Code	Sub-code(s)	Aspects Perceived As Positive ($\uparrow$) And Negative ($\downarrow$)	Quotes	Suggestion for Improvement
**Design & Format**	Layout, colours, presentation text	$\uparrow$ Friendly layout and colours	*‘So I think the design is good. I think it’s friendly and clear’. (ID14)*	More vivid colours and visuals to enliven the text
		$\downarrow$ Monotonous (a lot of text, pastel colours)	*‘So the size of the text is not too small, I don’t think so. But it’s a lot of text’. (ID05)* *‘[…] I have the impression that it could be depicted a bit more vividly, so that it doesn’t look completely uniform’. (ID09)*	
	Graphics, Structure and Info Boxes	$\uparrow$ Clear and comprehensible graphics $\uparrow$ Clear structure of content $\uparrow$ Info boxes as a preparation for physician–patient-talks	*‘And I believe, […] that if patients have the possibility to read things again or to understand them again on the basis of the graphics, some of which are very successful, or maybe even come to the conclusion, oh, I didn’t even ask that, I would perhaps like to ask that again, then that would probably be a super helpful tool’. (ID02)*	No suggestions for improvement based on mainly positive opinions
	Volume (number of pages)	$\uparrow$ Volume shows importance	*‘[…] I think it shows patients that they are taken seriously. […] People want patients to know what disease they have, what options they have, who can support them’. (ID05)*	Individual formats (e.g. chapters in separate brochures, unlock chapters step-by-step in online formats)
		$\downarrow$ Majority: Overwhelming volume	‘*Well, I’m talking about the volume. They [the patients] panic when I show them such bulky brochures’. (ID20)*	
	Format	$\uparrow$ Participants prefer brochure-format $\uparrow$ Age might be a factor in preference, as younger patients might prefer PDF and older patients print	*‘We can put something directly in the patient’s hand. This is a slightly more direct way than just giving them the link’. (ID16)*	
		PDF $\downarrow$ Limited access (problematic for patients without Internet access or those with little experience) $\downarrow$ Reading on screen may be exhausting because patients experience fatigue during treatment	*‘In rural areas we have the patient groups, let’s say […] 65 years old and upwards […], who are not familiar with social media or Internet […]’. (ID19)*	
**Comprehensibility**	Wording	$\uparrow$ Plain and objective language $\uparrow$ Restricted use of medical words	*‘In my opinion a lot of effort is being made to use plain language in order to make it understandable for laypeople. That means to use as few foreign medical words as possible […]’. (ID16)*	Wording of ‘Patientenleitlinie’ (PVG) Patient information brochure
		$\downarrow$ Wording ‘Patientenleitlinie’(PVG): does not amplify the original meaning of PVGs	*‘So, I think it’s good because it’s called patient guideline. […] However, a patient information brochure sometimes hits it a little bit better’. (ID04)*	
	Recommendations	$\uparrow$ Italic font in text	*‘[…] They [recommendations] are written in italics and if I remember correctly, it also says somewhere in the introduction how they are linked to the CPG’. (ID01)*	Put recommendations in bold print
		$\downarrow$ Difference in grading may be hard for patients to understand $\downarrow$ Hard to remember the definition of recommendations $\downarrow$ Italic font	*‘But I think that not all patients on page 40, when it says “should”, still know what that means. I know that because I am used to reading long texts’. (ID03)* *‘But in italics, now I see it. It’s down there, yes. Doesn’t stand out so much’. (ID04)*	
**Content**	Saturation of information	$\uparrow$ Most important aspects	*‘So in terms of content, I think it’s very, very good’. (ID04)*	Living PVGs to bring information up to date
		$\downarrow$ Missing content: information about self-care (e.g. breathing exercises), effects of sport exercises, treatment options for nausea, skin care, complementary medicine, information about long-term effects of treatments $\downarrow$ Not up to date (especially with regard to medications)	*‘What breathing exercises can I do to relax myself when I notice that panic comes up just before the examination. Or when I get the results of the laboratory examination, how can I calm myself down now? These are very simple, concrete techniques that can be experienced’. (ID06)* *‘But wait, if they want the latest information, so to speak, then PVGs [are] not the first choice’. (ID15)*	
**Trust**	In Content	$\uparrow$ Overall trust in content $\uparrow$ Recommend PVGs to patients, family and friends	*‘Since the PVG provides information from the CPG in common language, I find it [PVG] incredibly trustworthy’ (ID15)* *‘I could well imagine that I will recommend this [PVG] to patients more often.’ (ID19)*	
		$\downarrow$ Information on certain topics is not up to date (e.g. medications) $\downarrow$ Knowledge about methodical process in developing PVGs results in perceived inadequate content (e.g. complementary medicine)	‘*So the fact that I know which criteria have to be fulfilled so that they can be evidence-based at all, I am differentiated. Because I think many things do not have the chance to be validated due to such narrow criteria […]’. (ID03)*	

Abbreviations: $\uparrow$, Aspects perceived as positive from participants; $\downarrow$, Aspects perceived as negative from participants; CPG, clinical practice guideline; PVG, Patient version of clinical guideline

## Discussion

According to healthcare providers, PVGs seem to impact the relationship between patients and healthcare professionals and patients’ medical knowledge of their disease. The relevant aspects of the interviews with healthcare professionals are discussed below.

### Positive impact on patients‘ health literacy

The study results demonstrated that healthcare providers believe PVGs can positively influence patients’ health literacy (HL). For instance, healthcare providers mentioned that patients who were provided with PVGs were better informed. This is in line with previous and recent literature describing the positive impact of evidence-based information on patients with HL [8, 28, 29]. HL is described as the ability to ‘obtain, process, and understand basic health information and services in order to be able to make appropriate health decision’ [8]. It is heterogeneous among patients because its level depends on individual factors (e.g. education and culture) [8]. Two systematic reviews found that patients with low HL were more likely to obtain their health information from friends and family, television, or social media, whereas patients with high HL were more likely to turn to medical professionals [28, 29]. Additionally, a high level of HL has been associated with the ability to identify the trustworthiness and validity of health information [8, 28]. It is particularly important for healthcare providers to educate patients with low HL, and refer them to valid and evidence-based information, including that contained in PVGs. Nevertheless, PVGs are helpful for every patient and should be distributed regardless of their HL level.

Reliable and validated health information positively influences not only patients’ HL but also shared decision-making [30]. However, further research is needed to investigate PVG’s influence on patient knowledge and whether it increases informed decision-making.

### PVGs improve communication between healthcare providers and patients

In addition to PVGs’ capacity to support patients, this study showed that PVGs can function as useful tools for healthcare providers. According to participants, the use of PVGs in preparation for physician–patient conversations positively impacted general physician–patient communication in terms of structuring important topics and word choice. However, the preparatory role of patient information for healthcare providers in communication with patients has not yet been discussed in the recent literature, and further research is required in this area. Participants also positively highlighted the time-saving aspects of PVGs for medical appointments. Recent literature found no significant associations between the use of health information during medical appointments and time-saving effects owing to the poor quality of the included studies [31]. Thus, further research is required to address the impact of evidence-based health information on the duration of medical appointments.

PVGs might not only help healthcare providers prepare for communication with patients but also invite patients and healthcare providers to communicate more with patients about important aspects of treatment. Info boxes included in PVGs, such as questions before an operation, invite patients to engage in conversations with healthcare providers, which might facilitate more regular communication. Constant communication between healthcare providers and patients has been found to positively affect patient trust in healthcare providers, treatments, and health information [7, 32]. Additionally, patients endorse healthcare providers’ references to reliable and clear literature when time is taken to discuss and answer their questions [7]. Consequently, providing patients with PVGs and communicating about their content might improve patients’ trust in healthcare providers and, subsequently, the use and applicability of PVGs in patient care.

Furthermore, the results showed that patients do not always comprehend the intentions of the recommendations; specifically, that they are not mandatory for healthcare providers. According to the results of this study, patients’ misunderstanding of content can negatively affect general communication. Although the methods and intentions of the recommendations have already been described in PVGs, a clearer explanation and presentation are needed so that the content is fully comprehensible for patients. The presentation of PVG and explanation of its content could be part of a comprehensive inclusion in the healthcare provider’s communication with patients. Further research is needed on how the PVG can be actively used by healthcare providers in their communication with patients, e.g. through didactic training.

### Limited awareness of PVGs among healthcare providers

Naturally, in order to hand out PVGs to patients, healthcare providers must first know that they exist. Although healthcare providers see themselves as some of the main providers of PVGs for patients, their knowledge of the existence of PVGs remains limited. Alternative information materials, such as patient information from the German Cancer Aid [33], are better known to healthcare providers and used more frequently in healthcare. However, even though the participants suggested ways to raise awareness of PVGs (e.g. automatic distribution of brochures in inpatient and outpatient settings, promotion on social media, and delivery through self-help groups), further research is needed to determine appropriate approaches. Brochure distribution in inpatient and outpatient settings involves significant organisational effort and logistic challenges, such as retraining staff and producing, storing, and mailing PVGs. However, according to the participants, hospitals and medical practitioners have already received a significant amount of information. Consequently, the additional PVGs may be overlooked or discarded. Furthermore, hospitals and medical practitioners should ensure that brochures are updated so that patients receive the most current information. Additionally, mentioning PVGs in newsletters of relevant institutions, medical congresses, or other public events might be good options for raising awareness of their existence. In addition to participants’ limited awareness, fear of hidden costs seemed to impact the limited dissemination of PVGs in healthcare. This barrier might be addressed by displaying the cost neutrality of PVGs more clearly. Policy-makers and PVG creators should consider efficient and cost-effective approaches to improve the awareness and dissemination of PVGs in healthcare.

### Dissemination of PVGs

Most participants favoured distributing PVGs around the time of diagnosis. This is in line with findings of a qualitative study, which found that oncological patients require relevant health information from a very early start [34]. Only one interviewed participant in our study recommended dissemination at a later stage (e.g. during treatment). Which is also in line with the international literature. According to a systematic review, especially around the time of diagnosis, patients are confronted with negative emotions such as fear and distress, which might hinder accurate understanding health information. Therefore, the authors suggest avoiding possible barriers (e.g. stress and anxiety) when distributing health information to patients [35]. Another systematic review found that patients prefer health information be provided after diagnosis (e.g. during treatment) or be on demand [7]. Although the results of the current study show that most healthcare providers favour distributing PVGs to patients at an early stage, patients’ individual circumstances should be considered. Consequently, patients’ mental states and desire for health information should be key factors for healthcare providers when distributing PVGs. Overall, coping mechanisms and need of information are highly individual, hence there is no one-fits-all solution for all patients.

### Individual perceptions of design and format diversity

The assessment of the colours, design, volume, and format of the PVGs was based on participants’ individual perceptions. Some favoured the colours used because they radiated calmness, while others suggested the use of vivid colours to emphasise content. However, healthcare providers preferred the printed version of PVGs over the PDF version because the haptic format serves as a good tool for interacting with patients. From the healthcare providers’ point of view, patients’ preferences regarding the format of PVGs are heterogeneous and individual because younger patients may favour the PDF format while older patients may prefer the printed version. This is not in line with the results of previous literature as the preferred format of health information (web-based or print) was not significantly associated with patients’ age [36, 37]. Nevertheless, it should be noted that younger patients use the internet significantly more frequently than older patients [36].

The volume of the printed versions of the PVGs was perceived as sufficient by some and overwhelming by others. To address the perceived overwhelmingness, the content of printed PVGs can be produced in a staggered manner. Chapters can be issued in separate brochures. However, publishing the content of PVGs in separate brochures would not necessarily increase the awareness or dissemination of PVGs and may decrease clinics or practices’ willingness to order or store them. Further research is needed to address possible ways to individualise the formats of PVGs to include patient-relevant content without exaggerating the volume of PVGs and provoke overwhelmingness. One solution could be individualisation in the digital context. PVGs as apps could support individualisation by showing users only selected content or by changing the language of the content (e.g. foreign language or plain language). This could address target groups that are deterred from reading the PVGs due to the high volume, a language barrier, or an intellectual barrier. Moreover, representatives from patient organisations are involved in the development of PVGs and published PVGs can be evaluated via a feedback form included in the PVG. These channels can be used to adapt the PVGs to the needs of practice.

### Missing up-to-datedness of content

Missing up-to-date information (e.g. medications) limits participants’ trust in the content. Living CPGs may be an option to update content more frequently [27, 38]. Because PVGs are based on CPGs, their status can also be converted to a living status once the underlying CPG is adapted to a living CPG. On the one hand, living PVGs can positively impact dissemination and trust in content; on the other, they might add too much content to the already overwhelming volume of PVGs. In addition, the implementation of updated content would lead to a large number of updated versions of PVGs that would have to be published. Hence, living guidelines (CPGs and PVGs) involve a significant amount of organisational effort, which is time consuming and requires significant personnel deployment and monetary resources [38]. One solution may be the continuous updating of single chapters or specific content, such as information on medications [27, 39].

In addition to missing up-to-date information, participants noted that relevant content was missing in specific PVGs, such as information about patient self-care, nausea treatment, and complementary medicine (see Table 2). Some of the missing aspects were addressed in the specific PVG and might have been overlooked by participants. Furthermore, missing information can be found in additional PVGs (e.g. complementary medicine) provided by the GGPO.

### Limitations and strengths

The change in researcher personnel during the study period is a limitation of this study. The two researchers in charge of analysing the results (SW and JB) were not involved in planning the study or conducting the interviews. This was addressed through constant communication with the interviewer (MB).

One strength was the inclusion of a broad range of participants in terms of profession, thus representing a wide range of professions involved in the care of oncology patients. Furthermore, we included participants with a broad range of experiences and discussed different PVGs with different participants to gain an overview of the topic of PVGs as they differ in content and design. The results of this study should be considered in the context of further studies (qualitative interviews with oncological patients and mixed focus groups) that have also been conducted as part of the AnImPaLLO-project and have yet to be published. Together, they provide a comprehensive view of the topic of PVGs.

## Conclusion

Overall, participants had a generally positive impression of PVGs. PVG content and its comprehensibility positively impacted their applicability, especially in the context of physician–patient talks, while limited awareness and missing up-to-date information on specific content seemed to hinder the use and dissemination of PVGs in healthcare. Additionally, the use of alternative patient information appeared to be more common, with limited effects on the use and dissemination of PVGs. Although participants highlighted the time-saving aspects of PVGs in medical appointments, further research should address this discrepancy because the existing literature is of poor quality. Furthermore, policy-makers and PVG creators should consider efficient approaches to raise awareness of PVGs among healthcare providers, and improve their use and dissemination. To ensure successful implementation of PVGs in healthcare, training of healthcare providers on how best to communicate the contents of PVG to patients might be helpful. Moreover, the possible individualisation of formats and frequent updates of specific content based on living CPGs should be considered to improve the general applicability and use of PVGs in healthcare. In conclusion, further research is needed to investigate whether PVGs impact on patient knowledge and informed decision-making.

### Electronic supplementary material

Below is the link to the electronic supplementary material.

Supplementary Material 1

Supplementary Material 2

Supplementary Material 3

## Data Availability

The generated and analysed datasets are not publicly available due to the need to protect participants’ privacy and confidentiality. These datasets are available from the corresponding author upon request.

## Abbreviations

AWMF-IMWi: Scientific Medical Societies in Germany - Institute for Medical Knowledge Management
CPG: Clinical Practice Guidelines
GGPO: Office of the German Guideline Program in Oncology c/o German Cancer Society
HL: Health literacy
IOM: Institute Of Medicine
PVG: Patient version of clinical guideline
