# Supplementary material for: Use, applicability, and dissemination of patient versions of clinical practice guidelines in oncology in Germany: a qualitative interview study with healthcare providers
Source: BMC Health Serv Res. 2024 Mar 4;24:272. doi: 10.1186/s12913-024-10626-8 (PMC10913627; doi:10.1186/s12913-024-10626-8)
Supplement: Supplementary file 1 — Supplementary Material 1 [file 12913_2024_10626_MOESM1_ESM.pdf]

Table s1: COREQ checklist

Use, applicability and, dissemination of patient versions of clinical practice guidelines in oncology in Germany: A qualitative interview study with healthcare providers

Sarah Wahlen[1], Jessica Breuing[1], Monika Becker[1], Stefanie Bühn[1], Julia Hauprich[1], Nadja Könsgen[1], Nora Meyer[1], Susanne Blödt[2], Günther Carl[3], Markus Follmann[4], Stefanie Frenz[5], Thomas Langer[4], Monika Nothacker[2], Corinna Schaefer[6], Dawid Pieper[1,7,8]

[1] Institute for Research in Operative Medicine (IFOM), Witten/Herdecke University, Cologne, Germany
[2] Institute for Medical Knowledge Management c/o Philipps University Marburg, Association of the Scientific Medical Societies in Germany, Marburg / Berlin, Germany
[3] German Prostate Cancer Support Group, Bonn, German
[4] Office of the German Guideline Program in Oncology (GGPO), c/o German Cancer Society, Berlin, Germany
[5] Frauenhilfe Krebs Bundesverband e.V., Bonn, Germany
[6] German Agency for Quality in Medicine, Berlin, Germany
[7] Faculty of Health Sciences Brandenburg, Brandenburg Medical School (Theodor Fontane), Institute for Health Services and Health System Research, Rüdersdorf, Germany
[8] Centre for Health Services Research, Brandenburg Medical School (Theodor Fontane), Rüdersdorf, Germany

Consolidated criteria for reporting qualitative studies (COREQ) [1]

Table s1: COREQ: 32-item checklist

**Domain 1: Research team and reflexivity**	
Personal characteristics	
1. Interviewer/facilitator<br>Which author/s conducted the interview or focus group?	2.3 Data Collection<br>*“The data were collected via telephone interview by one author (MB), female researcher and, at the time, doctoral candidate at Witten/Herdecke University. The interviewer was trained in advance in qualitative interviews and analysis.”*
2. Credentials:<br>What were the researcher’s credentials? E.g. PhD, MD	
3. Occupation:<br>What was their occupation at the time of the study?	
4. Gender:<br>Was the researcher male or female?	
5. Experience and training:<br>What experience or training did the researcher have?	
Relationship with the participants	
6. Relationship established:<br>Was a relationship established prior to study commencement?	2.3 Data Collection<br>*“The first contact between the participants and the interviewer occurred prior to the interviews when detailed information about the study (background, duration of the interview, and intention to publish the results), along with privacy statements, was provided to each participant. There was no relationship between the interviewer and participants”*
7. Participant knowledge of the interviewer:<br>What did the participants know about the researcher? e.g. personal goals, reasons for doing the research	*2.3 Data collection*<br>*“The participants only knew she was a researcher at Witten/Herdecke University.”*
8. Interviewer characteristics:<br>What characteristics were reported about the interviewer/facilitator? e.g. Bias, assumptions, reasons and interests in the research topic	2.3 Data collection<br>*See text from rows 6 and 7.*
**Domain 2: study design**	
Theoretical framework	

9. Methodological orientation and Theory:<br>What methodological orientation was stated to underpin the study? e.g. grounded theory, discourse analysis, ethnography, phenomenology, content analysis	2.5 Data analysis<br>*"Therefore, the analysis was performed by two other authors (SW and JB). MAXQDA software (version 2022) was used to perform the interview analysis according to Mayring's content analysis method"*
Participant selection	
10. Sampling:<br>How were participants selected? e.g. purposive, convenience, consecutive, snowball	2.2 Recruitment<br>*"Participants were recruited via an online survey to analyse their awareness and the role of PVGs in oncology. The survey was conducted between April and June 2021 by the AWMF-IMWi. Additionally, project partners published calls for study participation via the Internet (e.g. newsletters, websites, social media) or flyers. After creating a list of all existing centres using Microsoft Excel, we also contacted a randomised national sample of certified and non-certified oncology centres in Germany. A new numeration of the centres was created by assigning a random number to each centre using the RAND function, and the first 50 centres on the list were contacted. A central organisation (the German Cancer Society) certifies oncology centres and recognises inpatient and outpatient facilities that form a network (centre) to improve the treatment of oncology patients through cooperative efforts. Information on certification of oncology centres in Germany can be found on the OnkoZert website. Relevant hospital units of certified and non-certified centres (e.g. outpatient clinic, psycho-oncology) were contacted by telephone to recruit medical providers who were directly involved in patient care. If the telephone approach was unsuccessful or impossible, the relevant hospital units were contacted via email. Moreover, we asked the participants whether they could pass on information about the study to colleagues to recruit more participants (snowball recruitment method). Recruitment ended when saturation was reached, indicating no additional analytical themes."*
11. Method of approach:<br>How were participants approached? e.g. face-to-face, telephone, mail, email<br>Reasons?	2.3 Data Collection<br>*"Data were collected via telephone interviews with one author (MB), female researcher and, at the time, doctoral candidate at Witten/Herdecke University. (…) All interviews were conducted via telephone from October to December 2021 using a recording device."*
12. Sample size:<br>How many participants were in the study?	3 Results<br>*"The remaining 20 healthcare providers participated in the semi-structured telephone interviews."*
13. Non-participation:<br>How many people refused to participate or dropped out?<br>Reasons?	3 Results<br>*"Overall, 36 healthcare providers showed an interest in participating in the study. A total of 16 participants (44%) did not participate, either because interviewers were unable to reach interested healthcare providers or because they were no longer working in the field of oncology."*
Setting	

14. Setting of data collection:<br>Where was the data collected? e.g. home, clinic, workplace	*2.3 Data collection*<br>*"The interviewer joined the interview from the workplace or home office, and participants were free to choose a convenient place and timeframe. Consequently, the presence of other people cannot be ruled out."*
15. Presence of non-participants:<br>Was anyone else present besides the participants and researchers?	
16. Description of sample:<br>What are the important characteristics of the sample? e.g. demographic data, date	3 Results<br><br>Table 1
Data collection	
17. Interview guide:<br>Were questions, prompts, guides provided by the authors? Was it pilot tested?	*2.3 Data collection*<br>*"The interview guide was designed prior to conducting the interviews and consisted of three main sections: 1) general information, 2) general questions about PVGs, and 3) questions about specific PVGs, and was reviewed and modified by the project team (Supplement 2: interview guide). Two pre-test interviews were conducted, which did not result in any changes to the interview guide."*
18. Repeat interviews:<br>Were repeat interviews carried out? If yes, how many?	2.3 Data Collection<br>*"No repeat interviews were required."*
19. Audio/visual recording:<br>Did the research use audio or visual recording to collect the data?	*2.3 Data collection*<br>*"All interviews were conducted via telephone from October to December 2021 using a recording device."*
20. Field notes:<br>Were field notes made during and/or after the interview or focus group?	2.3 Data Collection<br>*"Field notes were not taken during the interviews."*
21. Duration:<br>What was the duration of the interviews or focus group?	3 Results<br>*"The average duration of the interviews was 34 minutes (range: 20–49 minutes)."*
22. Data saturation:<br>Was data saturation discussed?	2.2 Recruitment<br>*"Recruitment ended when saturation was reached, indicating no additional analytical themes."*
23. Transcripts returned:<br>Were transcripts returned to participants for comment and/or correction?	2.4 Data Processing<br>"*Participants were not asked to provide feedback on their findings or transcripts."*
**Domain 3: analysis and findings**	
Data analysis	
24. Number of data coders:<br>How many data coders coded the data?	2.5 Data analysis<br>*"Therefore, the analysis was performed by two other authors (SW and JB)."*
25. Description of the coding tree:<br>Did authors provide a description of the coding tree?	Supplement 3: data coding tree
26. Derivation of themes:<br>Were themes identified in advance or derived from the data?	2.3 Data collection<br>*"The interview guide was designed prior to conducting the interviews and consisted of three main sections: 1) general information, 2) general questions about PVGs, and 3) questions about specific PVGs, and was reviewed and modified by the project team (Supplement 2: interview guide)."*<br><br>2.4. Data analysis<br>*"The interviews were analysed in a two-step process. First, two authors (SW and JB) coded the five transcripts independently and met to discuss and reach a consensus on the a priori defined data codes.*

	Afterwards, they split the remaining sample of interviews and carried out the analysis independently with ongoing consultation meetings (deductive approach). Second, the final sample of data codes was split in half, and each author independently generated sub-codes for the selected data codes for the entire interview set. The sub-codes were presented, discussed, and agreed upon by the two authors during ongoing meetings (inductive approach). Further issues that arose during the independent coding process were discussed by the two authors during consultation meetings. Subsequently, the coding framework with the final categories and sub-categories was reviewed by a team of authors (SW, JB, SBl, and MN), and minor editorial modifications were made."
27. Software:<br>What software, if applicable, was used to manage the data?	2.5 Data analysis<br>*"MAXQDA software (version 2022) was used to perform the interview analysis according to Mayring's content analysis method."*
28. Participant checking:<br>Did participants provide feedback on the findings?	2.4 Data Processing<br>*"Participants were not asked to provide feedback on their findings or transcripts."*
Reporting	
29. Quotations presented:<br>Were participant quotations presented to illustrate the themes / findings? Was each quotation identified? e.g. participant number	Varies quotations were presented within the results section.<br>Example: 3.1 Impact in healthcare<br>*"But if the patient who arrives with the guideline and says, "Now you have to do this and this", it can lead to a problematic relationship." (ID15)*
30. Data and findings consistent:<br>Was there consistency between the data presented and the findings?	Major themes were highlighted by the layout in the results section and numbering.<br>3. Results<br>• 3.1 Impact On Healthcare<br>• 3.2 Dissemination<br>• 3.3 Other Topics (incl. Table 2)<br>4. Discussion<br>• Positive impact on patients' health literacy<br>• PVGs improve communication between healthcare providers and patients<br>• Limited awareness of PVGs among healthcare providers<br>• Dissemination of PVGs<br>• Individual perceptions of design and format diversity<br>• Missing up-to-datedness of content<br>• Limitations and strengths
31. Clarity of major themes:<br>Were major themes clearly presented in the findings?	See headings of the results section:<br>3.1 Impact On Healthcare<br>3.2 Dissemination<br>3.3 Other Topics (incl. Table 2)
32. Clarity of minor themes:<br>Is there a description of diverse cases or discussion of minor themes?	Minor themes were presented in chapter 3.3 (Other topics) and discussed in the following (Discussion). Furthermore, Table 2 contains controversial and contrasting statements of participants.

1. Tong A, Sainsbury P, Craig J. Consolidated criteria for reporting qualitative research (COREQ): a 32-item checklist for interviews and focus groups. Int J Qual Health Care. 2007;19(6):349-57. Epub 20070914. doi: 10.1093/intqhc/mzm042. PubMed PMID: 17872937.
