# Supplementary material for: Use, applicability, and dissemination of patient versions of clinical practice guidelines in oncology in Germany: a qualitative interview study with healthcare providers
Source: BMC Health Serv Res. 2024 Mar 4;24:272. doi: 10.1186/s12913-024-10626-8 (PMC10913627; doi:10.1186/s12913-024-10626-8)
Supplement: Supplementary file 2 — Supplementary Material 2 [file 12913_2024_10626_MOESM2_ESM.pdf]

Additional file 2: Interview guide

Use, applicability and dissemination of patient versions of clinical practice guidelines in oncology in Germany - a qualitative interview study with health care providers

M.Sc. Sarah Wahlen[1] (sarah.wahlen@outlook.de), Dr. Jessica Breuing[1] (Jessica.breuing@uni-wh.de), M.Sc. Monika Becker[1] (m.becker@md-bund.de), Dr. Stefanie Bühn[1] (stefanie.buehn@klimawandel-gesundheit.de), M.A. Julia Hauprich[1] (juliahauprich@web.de), Dr. Nadja Könsgen[1] (Nadja.koensgen@uni-wh.de), M.Sc. Nora Meyer[1] (Nora.meyer@uni-wh.de), Dr. Susanne Blödt[2] (bloedt@awmf.org), Günther Carl[3] (guenther.carl@prostatakrebs-bps.de), Dr. Markus Follmann[4] (follmann@krebsgesellschaft.de), Stefanie Frenz[5] (s.frenz@frauenselbsthilfe.de), Dipl.-Soz. Wiss. Thomas Langer[4] (langer@krebsgesellschaft.de), Dr. Monika Nothacker[2] (nothacker@awmf.org), M.A. Corinna Schaefer[6] (schaefer@azq.de), Prof. Dr. Dawid Pieper[1,7,8] (Dawid.Pieper@mhb-fontane.de)

[1] Institute for Research in Operative Medicine (IFOM), Witten/Herdecke University, Cologne, Germany
[2] Institute for Medical Knowledge Management c/o Philipps University Marburg, Association of the Scientific Medical Societies in Germany, Marburg / Berlin, Germany
[3] German Prostate Cancer Support Group, Bonn, German
[4] Office of the German Guideline Program in Oncology (GGPO), c/o German Cancer Society, Berlin, Germany
[5] Frauenhilfe Krebs Bundesverband e.V., Bonn, Germany
[6] German Agency for Quality in Medicine, Berlin, Germany
[7] Faculty of Health Sciences Brandenburg, Brandenburg Medical School (Theodor Fontane), Institute for Health Services and Health System Research, Rüdersdorf, Germany
[8] Centre for Health Services Research, Brandenburg Medical School (Theodor Fontane), Rüdersdorf, Germany

Corresponding author: Sarah Wahlen (sarah.wahlen@outlook.de), Tel. +49 221 9895747

AnImPaLLo – Interview guide healthcare providers

Hello Mrs/Mr (...)

my name is (...) from the University of Witten/Herdecke (in Cologne). I am very pleased that you are participating in our interview.

Do you have any questions or comments before we start?

First of all, I would like to point out that there are no right or wrong answers.

It's all about your opinion and experience. Don't be surprised if some questions sound similar. Please say if you do not understand something or if you do not want to talk about something.

As you know, we are recording the interview. You have already given your consent in writing. Now I would like to ask you again so that we have the consent on the tape. I'm going to turn on the device now [turn on recorder

"Do you agree with the recording?" [tick yes/no]

Introductory questions

Now, in the first part of our conversation, I will ask you a few questions about yourself.

- [note gender] I note to myself you are female/male, that is correct so far?
- Would you tell me how old you are?
- Would you please tell me your highest level of education?
- You are a doctor/nurse/psychotherapist.
  How long have you been working as a doctor/nurse/psychotherapist?
- How long have you been working in the field of oncology?
- Since when do you mainly care for patients with [oncological disease]?
- Do you work with self-help groups / patient organizations? (IF YES, which ones?)

- **<u>Patient guidelines in general (knowledge, use)</u>**

To begin, I would first like to ask you a few general questions about PatLL and your experience with it.

- Can you describe to me in your own words what you understand by a patient guideline / what do you expect from a PatLL / what would you expect from a PatLL?
- Tell us about your experience with PatLL so far. / How do you use PatLL?
    - Are there differences between patients who know a PatLL and those who do not?
    - EXAMPLES
- Did you know PatLL of the oncology guideline program beforehand?

  IF YES/NOT MENTIONED:

    - How did you become aware of the PatLL?
    - Are PatLL provided in your clinic?

      IF YES

        - In what form?
        - IF NOT SAID: Do you point this out to your patients?

          IF YES: Do you additionally point out other information to your patients? Which?

          IF NO: Do you point your patients to other information? Which?
    - For which target group do you think the PatLL are primarily designed?

      Who are the PatLLs aimed at? Who can use them?

- **<u>Specific questions concerning the PatLL [xy]</u>**

In the following, I would like to ask you specific questions about PatLL [xy]. This also involves how you assess various aspects for the target groups / patients.

- Tell us, what did you like about PatLL, what did you miss?

  IF aspects are not mentioned or, if necessary, follow up when aspects are mentioned.

  - **Design**
    - ➢ What do you think of the design of the PatLL?
  - **Illustration**
    - ➢ *Assessment of readability for patients (target group)*
    - ➢ How do you rate the presentation of the text (e.g. font size and color, spacing, background color)?
    - ➢ What do you think of the language used? (if necessary, examples: length of sentences, personal address)
    - ➢ Do you have any suggestions to improve the readability of the PatLL (even more)?
  - **structure**
    - ➢ Can you say something about the structure of the PatLL? How do you find the structure of the PatLL?
    - ➢ In your opinion, are there enough pictures and graphics shown?
    - ➢ According to the S3-LL, the PatLL contains recommendations (German: “soll”, “sollte”, “kann”) that are shown in italics.

      Did you perceive the recommendations in the PatLL?

      What do you like about the presentation of the recommendations, what not?

      Do you have any suggestions for improvement?
  - **Comprehensibility (ask for examples of which sections are good, which are less so).**
    - ➢ Finden Sie die Patientenleitlinie verständlich für Pat. / andere Nutzer?
    - ➢ What do patients report?
    - ➢ Are there aspects that are not so understandable from your point of view? Can you give examples?

    IF NOT MENTIONED: In your opinion, how comprehensible are the recommendations for patients / other target groups?

    Importance of recommendation strengths; how helpful are "can do" recommendations?

- **format**
  - ➢ How would you rate the format of the PatLL?
  - ➢ In your opinion, which formats are suitable for different patients (target groups)?
  - ➢ How would you rate the scope of PatLL? for different patients (target group)?
  - ➢ What suggestions do you have for improvement?
  - ➢ IF NOT NOTED: follow up: Would individualized formats of PatLL xy be useful? (adapted to the respective target group, e.g. young / old) how feasible? What does individualized mean? *Example QR codes in brochure / PDF*

Trustworthiness

- How trustworthy do you assess the information in this PatLL? Can you give reasons for your assessment?
  - Would you recommend the PatLL to others?
  - IF YES, why? IF NO: Why not?

Relevance and importance of the information for the target groups

- In your view, to what extent does the information in this PatLL meet the information needs of different patients / different target groups
  - Which information did you find particularly important?
  - Which information was missing?
  - Which information could you rather do without? / are too much

Meaning of PatLL

- How could the PatLL be used [Pick up information from above if necessary].
  What role do you think the PatLL could play in managing with the disease?
  Where specifically could the PatLL help? Do you think it makes your job easier or harder if patients know the PatLL?
  Can the PatLL also lead to MISunderstandings?

  How do you think PatLL can be used by different target groups? (e.g. patients, relatives and care providers, different levels of education)?

IF NOT NOTED, possibly ask about the following aspects:

Communication between patients/relatives and providers, decision-making processes /SDM, management of the disease, analog information for providers and patients (unique selling point).

In your view, would it be useful for the PatLL to include additional decision support? [explain if necessary: Instruments that list advantages and disadvantages of a therapy, as well as provide information on alternatives, examples decision tables, computer programs]

- How do you rate PatLL compared to other available information?
  WHY? Difference to blue guides?

Time

When should the patient receive / be informed about the PatLL? By whom?

Enabling and hindering factors for the dissemination / use of PatLL.

- What do you think could improve the dissemination/use of PatLL and what could make it more difficult?

I have now reached the end of the interview. My questions have been answered well. Is there anything else you would like to add to the interview?

I now switch off the recording device. [Switch off recording device]

Would you like to be informed about the results of our study? If yes: How would you like to be contacted?

IF active in breast, colorectal or prostate cancer:

One last question: We plan to conduct focus groups following the interviews. These will probably take place in spring/summer 2022 in NRW. Are you interested in participating in such a focus group together with other health care providers and patients?

Then thank you very much for your participation, your opinion and your time!
