# Supplementary material for: Use, applicability, and dissemination of patient versions of clinical practice guidelines in oncology in Germany: a qualitative interview study with healthcare providers
Source: BMC Health Serv Res. 2024 Mar 4;24:272. doi: 10.1186/s12913-024-10626-8 (PMC10913627; doi:10.1186/s12913-024-10626-8)
Supplement: Supplementary file 3 — Supplementary Material 3 [file 12913_2024_10626_MOESM3_ESM.pdf]

Additional file 3: Data Coding System

Use, applicability and dissemination of patient versions of clinical practice guidelines in oncology in Germany - a qualitative interview study with health care providers

M.Sc. Sarah Wahlen[1] (sarah.wahlen@outlook.de), Dr. Jessica Breuing[1] (Jessica.breuing@uni-wh.de), M.Sc. Monika Becker[1] (m.becker@md-bund.de), Dr. Stefanie Bühn[1] (stefanie.buehn@klimawandel-gesundheit.de), M.A. Julia Hauprich[1] (juliahauprich@web.de), Dr. Nadja Könsgen[1] (Nadja.koensgen@uni-wh.de), M.Sc. Nora Meyer[1] (Nora.meyer@uni-wh.de), Dr. Susanne Blödt[2] (bloedt@awmf.org), Günther Carl[3] (guenther.carl@prostatakrebs-bps.de), Dr. Markus Follmann[4] (follmann@krebsgesellschaft.de), Stefanie Frenz[5] (s.frenz@frauenselbsthilfe.de), Dipl.-Soz. Wiss. Thomas Langer[4] (langer@krebsgesellschaft.de), Dr. Monika Nothacker[2] (nothacker@awmf.org), M.A. Corinna Schaefer[6] (schaefer@azq.de), Prof. Dr. Dawid Pieper[1,7,8] (Dawid.Pieper@mhb-fontane.de)

[1] Institute for Research in Operative Medicine (IFOM), Witten/Herdecke University, Cologne, Germany
[2] Institute for Medical Knowledge Management c/o Philipps University Marburg, Association of the Scientific Medical Societies in Germany, Marburg / Berlin, Germany
[3] German Prostate Cancer Support Group, Bonn, German
[4] Office of the German Guideline Program in Oncology (GGPO), c/o German Cancer Society, Berlin, Germany
[5] Frauenhilfe Krebs Bundesverband e.V., Bonn, Germany
[6] German Agency for Quality in Medicine, Berlin, Germany
[7] Faculty of Health Sciences Brandenburg, Brandenburg Medical School (Theodor Fontane), Institute for Health Services and Health System Research, Rüdersdorf, Germany
[8] Centre for Health Services Research, Brandenburg Medical School (Theodor Fontane), Rüdersdorf, Germany

Corresponding author: Sarah Wahlen (sarah.wahlen@outlook.de), Tel. +49 221 9895747

1 Coding Tree

Content	Category	Subcode	Rule	Category specification
General information (black)	assigned patient version	/	One word	
	Clinical Indication of patient	/	One word	
	gender	Female	One word	
		Male		
	age	/	Just numbers	In years
	education	ISCED >5	One word	OECD, E.U., UNESCO-UIS. ISCED 2011 Operational Manual Guidelines for classifying national education programmes and related qualifications. 2015<br>Autorengruppe Bildungsberichterstattung, L.-I.f.B.u.B. Bildung in Deutschland 2020 - Ein indikatorengestützter Bericht mit einer Analyse zu Bildung in einer digitalisierten Welt. 2020
		ISCED 3/4		
		ISCED <3		
	Profession	Psychotherapist/Psychooncologist	One word	Actual occupation, not learned profession (e.g. a psychiatrist who works as a psycho-oncologist is referred as such and not as a physician)
		Physiotherapist		
		Nurse/medical assistant		
		Physician		
		Nurse		
	professional experience in oncology		Just numbers and unit	time in years
	professional experience in oncology with specific indications		Context	If experience refers to chosen patient version (specific cancer indication)
	interaction with support groups	No	Context	Yes: name of support group
		Yes (name of the support group)		
	Type of institution	Private practice	One word	Private practice/ clinic with certification/ clinic without certification
		clinic with certification		
		clinic without certification		

General questions about patient version (blue)	definition patient version		Context	Aim/Goal/definition of patient version (individual perception)
	expectation patient version		Context	expectation of patient version (individual perception)
	experience with patient version y/n	If yes:<br>Which patient version was already known	Context	
		If yes:<br>provision of patient version by clinic or practice		No/online [pdf]/print
		If yes:<br>Referral of patient version to patients		Which additional/other information sources were recommended to patients
		If yes: Referral of other information sources to patients		e.g. blue brochures
		If yes: patients use of patient version		Does the patient use the patient version (and how frequently)
	differences in patients related to the use of patient version		Context	Differences in target groups related to the use of patient version
	target group		Context	Definition of target population / Who does the patient version address
General judgement (brown)	what did you like about the patient version		Context	General perception of the patient version
	what did you not like about the patient version			
Design and presentation (red)	design of the patient version		Context	Overall impression (e.g., Color, size, pictures)
	presentation of the text			color, size, spacing
	language			length of phrases, addressing
	structure of the patient version			Structure of content, topics
	images and graphic arts			graphic realization, pictograms
	Suggestions for improvement (design)			Any suggestions or comments for improvement (design), How can suggestions/recommendations be implemented

	recommendations - perception			Presentation, format
	recommendations - pros and cons			Pro/Con of the usage of recommendations in patient version
	suggestions for improvement (recommendations)			Any suggestions or comments for an improvement of the design (reccomendations),<br>How can suggestions/recommendations be implemented?
Comprehension (grey)	comprehensibility - own assessment		Context	General understandability, readability, simplicity
	comprehensibility of recommendations for patients		Context	Understandability, readability, simplicity of the recommendations
	Comprehensibility – patients assessment			Perceptions/Comprehensibility of the recommendations from patient's point of view
Format (green)	presentation format - own assessment		Context	General perception of the presentation format
	presentation format - target group		Context	Which format/design is best for each target group?
	volume/quantity of patient version		Context	What is your opinion about the scope/extent of the patient version for different target groups?
	suggestions for improvement (format)		Context	Pro/con implementation
Trustworthiness	trustworthiness in information		Context	Are the information trustworthy
	recommend patient version y/n		Context	Would interview partner recommend the patient version
Content (yellow)	saturation of information needs		Context	Extent/amount of information
	important information		Context	Are the most important information included / which are the most important information
	missing information		Context	Additional information needed?
	dispensable information		Context	Which information can be shortened/cancelled
	Comment or suggestion for improvement (content)		Context	Any comment or suggestions for improvement in content
	impact of patient versions in healthcare		Context	Which impact did the patient version have / Which impact will the patient version have on healthcare provision

Impact of patient version (orange)	impact of additional decision aid		Context	Is additional decision aid needed? What impact may decision support have? (Pro/Con)
	impact of narrative			Is additional narrative needed? What impact may narrative have? (Pro/Con)
	impact compared to other information sources		Context	How does the patient version differ from other information sources e.g. blue brochures from the German Cancer Aid
Dissemination of patient version to the patient (turquoise)	Timing		Context	Ideal/wrong time to hand over the patient version<br>When does/should the patient get the patient version
	influencing factors on dissemination of patient versions	Barriers	Context	Are there any existing influencing factors which function as barriers or facilitators in the dissemination of patient versions
		facilitators		
	Comment or suggestions for improvement (dissemination)		Context	suggestions to improve dissemination/usage
Perception of specific topics	Perception of psychooncology		Context	Perception of the patient / Perception of psychooncologist about the patient’s treatment in phsycooncology (with/without patient version)
	Perception of palliative care		Context	Perception about the description of palliative care in patient version

Only the context is coded without the corresponding question, unless the context does not allow any conclusion about the underlying question.
